# Application of Anlotinib Combined With Neoadjuvant Chemotherapy in Primary EWS/PNET of Lung: A Case Report

**DOI:** 10.3389/fonc.2022.822469

**Published:** 2022-04-21

**Authors:** YH Fan, HX Ma, SP Guo, Y Chen, SP Zhang

**Affiliations:** ^1^ Department of Thoracic Surgery, Shanxi Province Cancer Hospital/Shanxi Hospital Affiliated to Cancer Hospital, Chinese Academy of Medical Sciences/Cancer Hospital Affiliated to Shanxi Medical University, Taiyuan, China; ^2^ Department of Pathology, Shanxi Province Cancer Hospital/Shanxi Hospital Affiliated to Cancer Hospital, Chinese Academy of Medical Sciences/Cancer Hospital Affiliated to Shanxi Medical University, Taiyuan, China; ^3^ Department of Operating Room Nursing, Shanxi Province Cancer Hospital/Shanxi Hospital Affiliated to Cancer Hospital, Chinese Academy of Medical Sciences/Cancer Hospital Affiliated to Shanxi Medical University, Taiyuan, China

**Keywords:** ewing’s Sarcoma, pulmonary, Anlotinib, Neoadjuvant chemotherapy, multi-targeted receptor tyrosine kinase

## Abstract

Primary pulmonary EWS/PNET(PPES) is extremely rare and is associated with a poor prognosis. Tumor angiogenesis plays an important role in tumor, so it has become a hot topic in molecular targeted therapy. Anlotinib is a new oral small molecular multi-targeted receptor tyrosine kinase (RTK) inhibitor. This report describes a 20 year-old man with PPES. After 4 neoadjuvant chemotherapy cycles (VACwith alternating IE) combined with anlotinib, the left total pneumonectomy was performed. Then maintenance anlotinib monotherapy was continued, no sign of recurrence to date as an outcome. To our knowledge, this is the first demonstration of anlotinib combined with neoadjuvant chemotherapy efficacy in PPES.

## Introduction

Primary pulmonary Ewing’s sarcoma (EWS)/Primitive neuroectodermal tumor (PNET) is a kind of extremly rare and highly invasive tumors, which belongs to the Ewing sarcoma family of tumors. The first case of Primary EWS/PNET of lung was described by Hammar in 1989 (1), there were reported less than 40 cases in literatures (1–8). Primary pulmonary EWS/PNET (PPES) occurs in all age with a peak between 20-30 years old, mostly in children and adolescents, without gender difference. The symptoms of PPES are non-specific as same as other malignant tumor in chest, which is usuarlly misdiagnosed as lung cancer upon preliminary diagnosis. Current managements including surgical, chemotherapy and radiotherapy. The tumors are usually insensitive to conventional chemotherapy, and large doses of drug can lead to serious toxic and side effects, such as neutropenia, infectious complications and thrombocytopenia. Anlotinib is a novel oral small molecular ereceptor tyrosine kinase inhibitor that targets VEGFR1, VEGFR3, VEGFR2/KDR, PDGFRA,C-kit and FGFRs (9). The recent studies showed that the combination of chemotherapy and anlotinib significantly improved survival and was well tolerated in patients with advanced/metastatic STS (10). We herein reports a case of PPES treated with anrotinib combined with neoadjuvant chemotherapy and postoperative maintenance.

## Case Report

**Figure 1:**
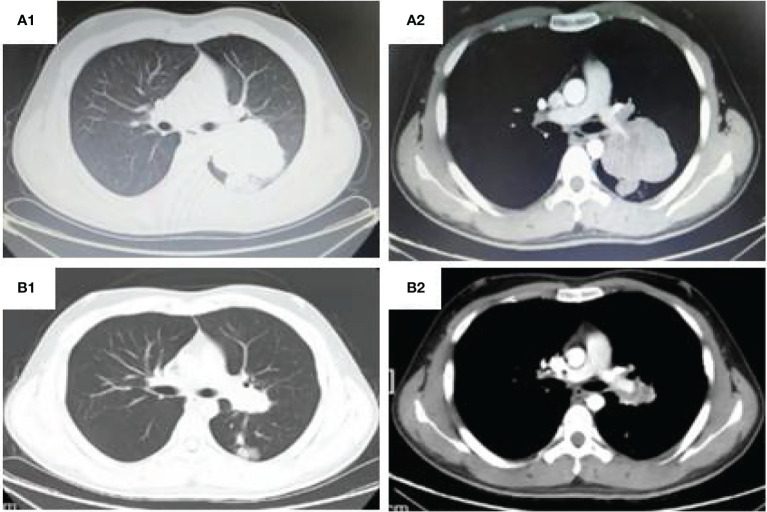
CT thorax: **(A1, 2)** (Shanxi Provincial Cancer Hospital,2021-02-03) shows a large irregular and well-defined border mass lesion measuring 7.7×6.3 cm with inhomogenous enhancement beside the left hilus pulmonis across the interlobar fissure and an enlarged mediastinal lymph node measuring 2.7×1.9 cm. **(B1, 2)**. (Shanxi Provincial Cancer Hospital,2021-05-12)Tumor was remarkable reduced to 2.5*3.4cm and the mediastinal lymph node was 1.2 cm.

**Figure 2:**
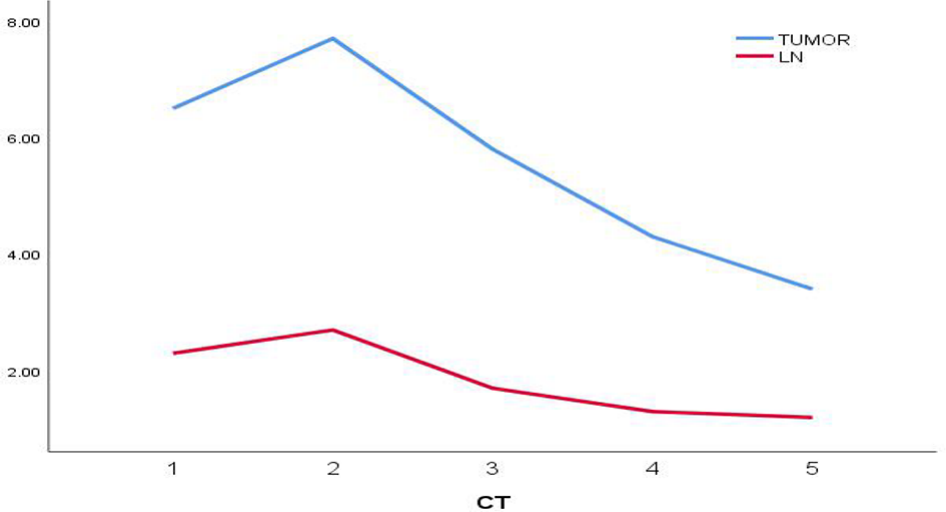
Pathological examination. **(A)** H&E×200/400 cell block shows small round tumour cells; **(B)** CD99(+)×200/400; **(C)** AE1/AE3(-)×200/400 **(D)** TTF-1(-)×200/400 **(E)** Ki67(appro. 80%+×400); **(F)** FISH shows EWSR1 gene rearrangement.

**Figure 3:**
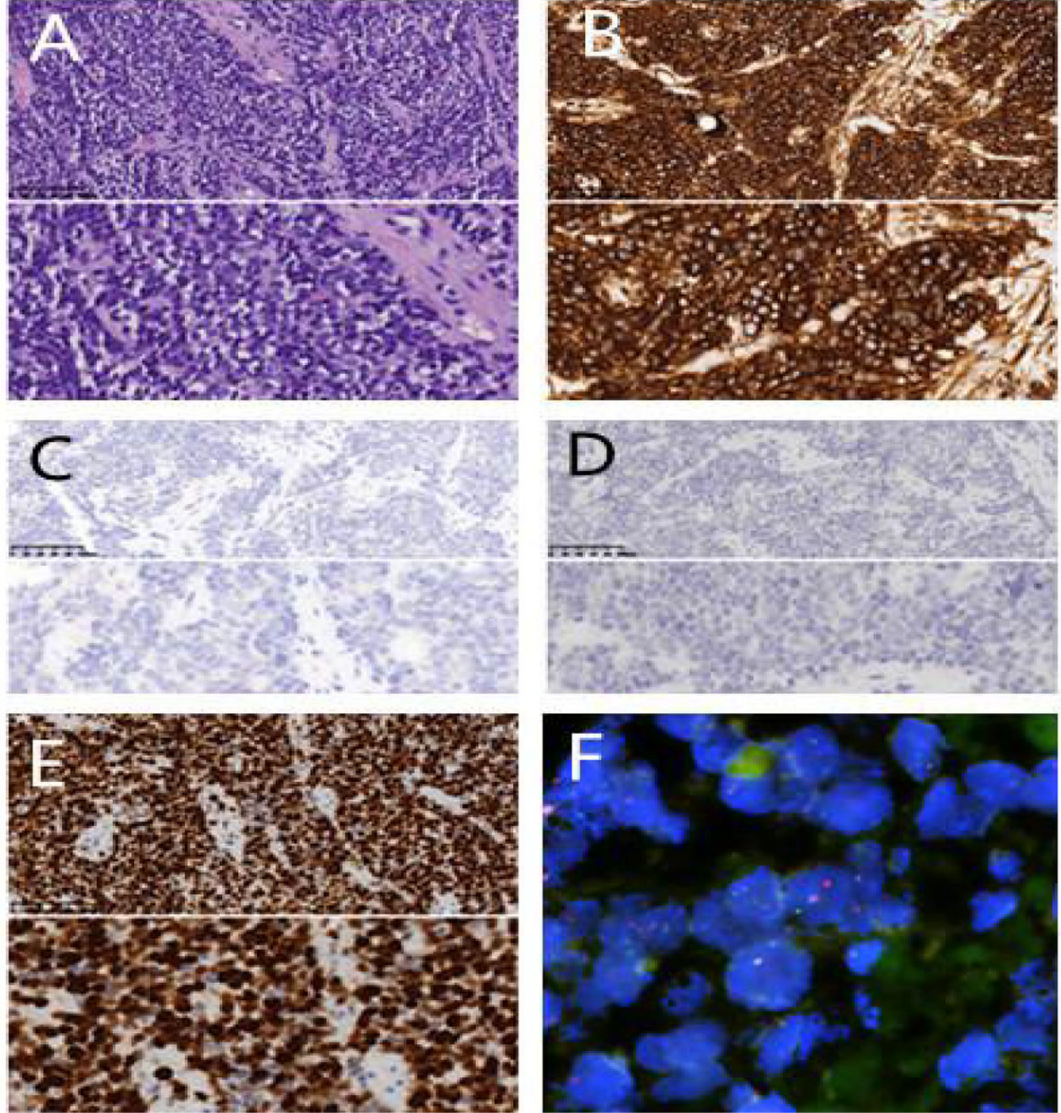
The evariation trend of the size of the tumor and the mediastinal lymph nod.

A 20 year old man was admitted to our department because of cough for 20 days. His family history was grandfather suffered from lung cancer. The findings of physical examination was no obvious abnormalities. A CT scan of thoracic at a local hospital showed a mass mesuring 6.5cm in the left lung on January 18^th^, 2021, which was irregular and well-defined border. Only 16 days later, CT thorax in our hospital revealed the mass increased to 7.7 x 6.3cm and an enlarged mediastinal lymph node measuring 2.7×1.9 cm. (**Figure 1**). Serum gastrin-releasing peptide and neuron specific enolase were104.68pg/mland13.65pg/ml. No intrabronchial lesion was found by bronchoscopy. Pathology from CT-guided percutaneous lung biopsy demonstrated small round cells in nests with abnormal nuclei. Immunohistochemically stained slides displayed that AE1/AE3(-), CD99(+), NSE(-), Ki67(appro. 80%+), TTF-1(-) and Syn(+). A fluorescent *in situ* hybridization (FISH) assay showed positive rearrangement of the ESWR1 gene **Figure 2**. Thus, a definitive diagnosis of PPES was made. The neoadjuvant chemotherapy regimen consisted of 4 alternating cycles of VAC (Vincristine 1.5mg/m2, ivgtt, D1, D8 and D15; Actinomycin D 1.5mg/m2, ivgtt, D1; Cyclophosphamide, ivgtt, 1.2g/m2,D1) and IE (Isocyclophosphamide 1.8g/m2, ivgtt, D1 -D5; Etoposide 100mg m^2^, ivgtt, D1 -D 5 )repeated every 3 weeks. The first 3 cycles were combined with anlotinib (12mg, PO,qd, D1-14 every 3 weeks). On March 4^th^, March 23^rd^ and May 12^th^, 2021, CT thorax re-examination showed that the size of the left mass was significantly decrease (5.8*4.7cm,4.3*2.7cm and 3.4 *2.5cm, respectively) (**Figure 3**). Left total pneumonectomy was performed on May 17^th^, 2021, and postoperative pathology showed that the tumor cells were degenerative and the interstitial fibrous tissue were proliferative and inflammatory cell were infiltrative, which conforms to the changes after treatment. None of lymph nodes metastasis was observed except one subcarinal lymph node. Whole Exome Sequencing found germ-line TP53 mutation NM_000546:exon4:c. C215G:p.P72R, and SKT11 system mutation NM_000455:exon9:c. G1274A:p.R425H. The patient refused chemotherapy after surgery because of the obvious myelosuppression due to the large toxic and side effect of chemotherapy drugs. Then maintenance anlotinib(12mg, PO, D1-14, every 3 weeks) monotherapy was continued, without disease progression for 5 months after operation.

## Discussion

The clinical symptoms of PPES are non-specific. Being the same as other malignant tumor occurring in chest, major clinical manifestations are cough, phlegm, chest pain or hemoptysis etc., which is often misdiagnosed as lung cancer upon preliminary diagnosis. Should the mass be large, it can cause corresponding mass effect and affect pleura, resulting in pleural effusion, causing Thoracic and back discomfort, dyspnea, fatigue and other symptoms (2–8). Such disease can occur at any age, mostly in children and adolescents, and peaks at the age of 20-30 years old, without gender difference.

CT characterization of Ewing’s sarcoma in the lung is still limited, and is usually described as (7, 11–13). 1st, The masses are of different sizes, being single or multiple, with clear boundaries and possible lobulation. Once found, the masses are usually huge (4-15cm). Mass in single lung is more common, but also can be seen in both lungs; 2nd, Density can be uniform or non-uniform, appro. 10% of tumors can be calcified; Upon enhancement, uniform enhancement, focal non-uniform enhancement or annular enhancement can be observed, and necrosis or hemorrhage can result in low or high density lesions. 3rd, The mass grows rapidly, and may possibly surround the pulmonary artery or pulmonary vein, or even infiltrate across the pulmonary lobe along the interlobar fissure. Large tumors may invade the mediastinum or displace mediastinal organs, and in severe cases, it may cause superior vena cava syndrome, where pleural thickening and malignant effusion can be seen. 4.12% may indicate lymph node metastasis.

PET-CT plays an important role in grading, prognosis and treatment response evaluation of sarcoma, and SUV value is correlated with tumor grading (14). Studies have found that in children’s Ewing’s sarcoma, sensitivity of PET-CT and CT are 77% and 100%, specificity are 71% and 56%, and accuracy are 73% and 79% respectively (15). PET-CT of Ewing’s sarcoma in lung often presents with large volume, smooth margins, and high fluorodeoxyglucose uptake, SUV MAX10-30.3. In addition, fluorodeoxyglucose positron emission tomography (PET) can help to rule out the possibility of pulmonary metastasis by extra-pulmonary Ewing’s sarcoma (4, 6–8, 16, 17).

Light microscopic performance: The tumor cells were small and round or oval, with with a prominent nucleus, scant cytoplasm and granular chromatin. The tumor cells were closely arranged, diffuse, foliated or nested. Homer Wright or Flexner Weinsteiner’s rosettes structure could be seen. Necrosis, hemorrhage, and cystic changes are common. Electron microscopy features include high nucleo-cytoplasmic ratio and aggregation of glycogenosome in cytoplasm.

Immunohistochemical (IHC) phenotypes showed characteristic expression of neural differentiation, with high expression of CD99, NSE, FLI-1 and Syn as relatively specific indicators for diagnosis. S-100 and CgA could also be expressed as positive in some patients, but lacked specificity. NKX2.2 was a marker with high sensitivity but low specificity, combination of high expression of CD99 and FLI-1 is a specific and sensitive immune index for the diagnosis of this disease.

Genetic characteristics of EWS/PNET is specific chromosomal translocation [5,16-17], which results in the fusion of the EWSR1 (22q12) gene with a member of the ETS transcription factor family: approximately 90% with FLI1 gene (11q24), which produces EWS-FLI1 fusion gene; 5%-10% with ERG gene (21q22). Rarely can the EWSR1 fuse with FEV (2q36), ETV1 (7p21) or ETV4 (E1AF;17q21). Such type of fusion gene is often used to diagnose pathological states, quantitative polymerase chain reaction (qPCR), EWS FLI1 and FLI1 EWS quantitative expression, to reveal the EWSR1 rearrangement by fluorescence *in situ* hybridization (FISH).

Due to its rarity, there is no standard treatment for primary ewing’s sarcoma of lung. Ewing’s sarcoma is characterized by strong invasiveness, rapid metastases and poor prognosis. The 5-year survival rate is only 25% (7). Current treatments include surgery, chemotherapy and radiation. The prognosis is mainly related to the degree of radical treatment. Standard first-line treatment consists of alternating chemotherapy with vincristine, doxorubicin, cyclophosphamide, ifosfamide, and etoposide. Chemotherapy can improve the survival rate after radical surgery (18). Tumor angiogenesis plays an important role in tumor, so it has become a hot topic in molecular targeted therapy. Especially in Soft-tissue sarcoma (STS), there was lack of driver mutations,due to the heterogeneity of tumor cells (19). Pazopanib is a multi-targeted receptor tyrosine kinase (RTK) inhibitor. Because of the results of the PALETTE study (20), pazopanib authorized for the treatment of STS by the FDA in 2012. Olaratumab is a human antiplatelet-derived growth factor receptor. In a phase Ib/II random trial of 133 patients with advanced STS,compared with doxorubicin alone, olaratumab in combination with doxorubicin improved PFS (6.6 months vs 4.1 months) and OS (26.5 months vs 14.7 months) (21). Anlotinib has more targets than the other RTK inhibitors, such as sorafenib,pazopanib, and sunitinib. It is conformed that anlotinib has satisfactory membrane permeability and absorption rates from drug preparation studies (9). The adverse events (AEs) of anlotinib were appeared in the different kinds of studies, however, most of the incidence were controllable, including hypertension, proteinuria, hand–foot skin reaction, hypothyroidism, elevated alanine aminotransferase, elevated aspartate transaminase, elevated total bilirubin, elevated triglyceride, etc (9, 10).

Wang etal. reported that ORR and DCR of first-line treatment (Anlotinib) was better than that of second-line and third-line treatment in advanced/metastatic STS patients who received chemotherapy combined with anlotinib plus anlotinib maintenance therapy. Therefore,the author considered the addition of anlotinib was significantly improve the efficacy (22). A study showed that 81.0% DCR was obtained in 21 patients with unresectable or metastatic STS with maintenance therapy with Anlotinib. It is therefore suggested that Anlotinib maintenance treatment is a promising approach with acceptable toxicity (23). Liu et al. reported that ORR was 40.7% and DCR was 81.5% in 27 advanced STS patients treated with Anlotinib and doxorubicin lipidosome combined with Anlotinib, and median progression-free survival was 7 months (24).

Recent studies have shown that anlotinib and pazopanib have been used in the treatment of PPES and achieve incredible results,but are not considered standard treatment (4, 8). Takigami reported that a case of PPES received neoadjuvant chemotherapy and total pneumonectomy, recurred 1.5 months after surgery, and achieved a progression-free survival of 5 months after taking pazopanib (4). Zhang reported that a case of PPES received 5 cycles of chemotherapy, but he did not benefit unambiguously. The patient started taking antirotinib,the tumor size was obvious decrease after 1 month (8). Unfortunately, no follow-up was not found inthe literature. Due to the reason that pazopanib was not in the market in China, and targets of anlotinib is more than pazopanib (9), anlotinib was selected for neoadjuvant chemotherapy combined with after MDT discussion.The size of the tumor was significant decrease and R0 resection was acheived. The patient refused chemotherapy after surgery because of the obvious myelosuppression due to the large toxic and side effect of chemotherapy drugs. Then maintenance anlotinib monotherapy was continued, till present, 6 months after surgery, no signs of recurrence was shown. However, the duration of anlotinib monotherapy has not been reported in literatures, and long-term follow-up will be required. To our knowledge, this is the first demonstration of anlotinib combined with neoadjuvant chemotherapy (VAC alternatingIE) efficacy in PPES.

## Data Availability Statement

The original contributions presented in the study are included in the article/supplementary material. Further inquiries can be directed to the corresponding authors.

## Ethics Statement

Written informed consent was obtained from the individual(s) for the publication of any potentially identifiable images or data included in this article.

## Author Contributions

ZS and CY: research design and guidance, data acquisition, analysis and evaluation of statistical results, critical revision of the manuscript, and study supervision. MH and FY: data acquisition, study concept and design, analysis and interpretation of data, statistical analysis, and drafting of the manuscript. GS: critical revision of the manuscript, and approved final submission. All authors contributed to the article and approved the submitted version.

## Conflict of Interest

The authors declare that the research was conducted in the absence of any commercial or financial relationships that could be construed as a potential conflict of interest.

## Publisher’s Note

All claims expressed in this article are solely those of the authors and do not necessarily represent those of their affiliated organizations, or those of the publisher, the editors and the reviewers. Any product that may be evaluated in this article, or claim that may be made by its manufacturer, is not guaranteed or endorsed by the publisher.
